# Supplementary material for: Long-term effects of THC exposure on reward learning and motivated behavior in adolescent and adult male rats
Source: Psychopharmacology (Berl). 2023 Mar 18;240(5):1151–67. doi: 10.1007/s00213-023-06352-4 (PMC10102061; doi:10.1007/s00213-023-06352-4)
Supplement: Supplementary file 1 — Supplementary file1 (DOCX 41 KB) [file 213_2023_6352_MOESM1_ESM.docx]

| Table S1: THC effects on bodyweight | | | | |
| --- | --- | --- | --- | --- |
| *First injection* | | | | |
|  | F value | d.f. | P value | η_p_^2^ |
| **Age** | **3230.49** | **1,36** | **<.001** | **.99** |
| Drug | .91 | 1,36 | .35 | .025 |
| Age x Drug | .036 | 1,36 | .85 | .001 |
| *Last injection* | | | | |
|  | F value | d.f. | P value | η_p_^2^ |
| **Age** | **1083.644** | **1,36** | **<.001** | **.97** |
| **Drug** | **20.31** | **1,36** | **<.001** | **.36** |
| Age x Drug | .061 | 1,36 | .81 | .002 |
| *Prior to testing* | | | | |
|  | F value | d.f. | P value | η_p_^2^ |
| **Age** | **14.73** | **1,36** | **<.001** | **.29** |
| Drug | 1.89 | 1,36 | .18 | .05 |
| Age x Drug | .37 | 1,36 | .55 | .01 |

**Table S1:** ANOVA results for Experiment 1 bodyweight data on the first and last injection day and prior to behavioral testing (dependent measure: grams). Age (Adult vs. Adolescent) and Drug (THC vs. vehicle) varied between subjects. See main text for details. Significant results (p < .05) indicated by **bold text**.

| Table S2: Instrumental training | | | | |
| --- | --- | --- | --- | --- |
|  | F value | d.f. | P value | η_p_^2^ |
| **Schedule** | **115.17** | **3,108** | **<0.001** | **.76** |
| Sched x Age | .78 | 3,108 | .77 | .01 |
| Sched x Drug | 1.86 | 3,108 | .14 | .049 |
| Sched x Age x Drug | .92 | 3,108 | .48 | .004 |
| Age | .45 | 1,36 | .51 | .012 |
| **Drug** | **5.22** | **1,36** | **.028** | **.13** |
| Age x Drug | .74 | 1,36 | .40 | .02 |

**Table S2:** Mixed ANOVA results for Experiment 1 instrumental training data (dependent measure: presses per minute). Schedule (FR1, RR5, RR10, RR20) varied within-subjects while Age (Adult vs. Adolescent) and Drug (THC vs. vehicle) varied between subjects. See main text for details. Significant results (p < .05) indicated by **bold text**.

| Table S3: Devaluation (original action-outcome relationships) | | | | |
| --- | --- | --- | --- | --- |
| *Extinction phase* | | | | |
|  | F value | d.f. | P value | η_p_^2^ |
| **Devaluation** | **57.97** | **1,36** | **<0.001** | **.62** |
| Deval x Age | .75 | 1,36 | .77 | .02 |
| Deval x Drug | .43 | 1,36 | .24 | .012 |
| Deval x Age x Drug | 1.11 | 1,36 | .48 | .03 |
| Age | .75 | 1,36 | .39 | .059 |
| Drug | .43 | 1,36 | .52 | .002 |
| Age x Drug | 1.11 | 1,36 | .30 | .043 |
| *Reinforced phase* | | | | |
|  | F value | d.f. | P value | η_p_^2^ |
| **Devaluation** | **104.86** | **1,36** | **<0.001** | **.74** |
| Deval x Age | .62 | 1,36 | .44 | .017 |
| Deval x Drug | 2.10 | 1,36 | .16 | .060 |
| Deval x Age x Drug | .018 | 1,36 | .90 | .000 |
| Age | .98 | 1,36 | .98 | .000 |
| Drug | 1.23 | 1,36 | .28 | .033 |
| Age x Drug | .55 | 1,36 | .46 | .015 |

**Table S3:** Mixed ANOVA results for Experiment 1 reward devaluation test data (separated for extinction and reinforced phases; dependent measure: presses per minute). Devaluation (Dev vs. Nondeval) varied within-subjects while Age (Adult vs. Adolescent) and Drug (THC vs. vehicle) varied between subjects. See main text for details. Significant results (p < .05) indicated by **bold text**.

| Table S4: Instrumental reversal training | | | | |
| --- | --- | --- | --- | --- |
|  | F value | d.f. | P value | η_p_^2^ |
| **Day** | **29.32** | **4,144** | **<0.001** | **.45** |
| Day x Age | .98 | 4,144 | .42 | .026 |
| Day x Drug | .51 | 4,144 | .73 | .014 |
| Day x Age x Drug | 1.26 | 4,144 | .29 | .034 |
| Age | .73 | 1,36 | .40 | .020 |
| Drug | 2.83 | 1,36 | .10 | .073 |
| Age x Drug | .00 | 1,36 | .98 | .000 |

**Table S4:** Mixed ANOVA results for Experiment 1 instrumental reversal training data (dependent measure: presses per minute). Day (1-5) varied within-subjects while Age (Adult vs. Adolescent) and Drug (THC vs. vehicle) varied between subjects. See main text for details. Significant results (p < .05) indicated by **bold text**.

| Table S5: Devaluation – extinction (reversed action-outcome relationships) | | | | |
| --- | --- | --- | --- | --- |
| *Extinction phase* | | | | |
|  | F value | d.f. | P value | η_p_^2^ |
| **Devaluation** | **8.98** | **1,36** | **0.005** | **.20** |
| Deval x Age | .65 | 1,36 | .43 | .018 |
| Deval x Drug | .49 | 1,36 | .49 | .013 |
| Deval x Age x Drug | .78 | 1,36 | .38 | .021 |
| Age | .55 | 1,36 | .46 | .015 |
| Drug | .42 | 1,36 | .52 | .012 |
| Age x Drug | 1.92 | 1,36 | .17 | .051 |
| *Reinforced phase* | | | | |
|  | F value | d.f. | P value | η_p_^2^ |
| **Devaluation** | **55.77** | **1,36** | **<.001** | **.61** |
| Deval x Age | .91 | 1,36 | .35 | .025 |
| Deval x Drug | .12 | 1,36 | .74 | .003 |
| Deval x Age x Drug | .01 | 1,36 | .92 | .000 |
| Age | 1.04 | 1,36 | .31 | .028 |
| Drug | .012 | 1,36 | .91 | .000 |
| Age x Drug | 1.48 | 1,36 | .23 | .040 |

**Table S5:** Mixed ANOVA results for Experiment 1 post-reversal training reward devaluation test data (separated for extinction and reinforced phases; dependent measure: presses per minute). Devaluation (Dev vs. Nondeval) varied within-subjects while Age (Adult vs. Adolescent) and Drug (THC vs. vehicle) varied between subjects. See main text for details. Significant results (p < .05) indicated by **bold text**.

| Table S6: Pre-degradation instrumental retraining | | | | |
| --- | --- | --- | --- | --- |
|  | F value | d.f. | P value | η_p_^2^ |
| **Day** | **33.35** | **1,36** | **<.001** | **.48** |
| Day x Age | 2.02 | 1,36 | .14 | .053 |
| Day x Drug | 2.06 | 1,36 | .13 | .054 |
| Day x Age x Drug | .91 | 1,36 | .41 | .025 |
| Deg | .37 | 2,72 | .55 | .010 |
| Deg x Age | .013 | 2,72 | .91 | .000 |
| Deg x Drug | .92 | 2,72 | .34 | .025 |
| Deg x Age x Drug | .39 | 2,72 | .54 | .011 |
| Day x Deg | .13 | 2,72 | .88 | .003 |
| Day x Deg x Age | .062 | 2,72 | .94 | .002 |
| Day x Deg x Drug | 2.07 | 2,72 | .13 | .054 |
| Day x Deg x Age x Drug | .073 | 2,72 | .93 | .002 |
| Age | 3.02 | 1,36 | .091 | .077 |
| **Drug** | **4.35** | **1,36** | **.044** | **.11** |
| Age x Drug | 0.00 | 1,36 | .99 | .000 |

**Table S6:** Mixed ANOVA results for Experiment 1 pre-degradation instrumental retraining data (dependent measure: presses per minute). Degradation (Deg vs. Nondeg) varied within-subjects while Age (Adult vs. Adolescent) and Drug (THC vs. vehicle) varied between subjects. See main text for details. Significant results (p < .05) indicated by **bold text**.

| Table S7: Degradation retraining (normalized to pre-deg baseline) | | | | |
| --- | --- | --- | --- | --- |
|  | F value | d.f. | P value | η_p_^2^ |
| **Degradation** | **60.76** | **1,36** | **<.001** | **.63** |
| Deg x Age | .051 | 1,36 | .82 | .001 |
| Deg x Drug | .032 | 1,36 | .86 | .001 |
| Day x Age x Drug | 1.43 | 1,36 | .24 | .038 |
| **Day** | **53.26** | **9,324** | **<.001** | **.60** |
| Day x Age | 1.07 | 9,324 | .38 | .029 |
| Day x Drug | .73 | 9,324 | .69 | .02 |
| Day x Age x Drug | .37 | 9,324 | .95 | .01 |
| Day x Deg | 1.87 | 9,324 | .056 | .049 |
| Deg x Day x Age | .65 | 9,324 | .76 | .018 |
| Deg x Day x Drug | .35 | 9,324 | .96 | .009 |
| Deg x Day x Age x Drug | 1.13 | 9,324 | .34 | .03 |
| Age | .29 | 1,16 | .59 | .008 |
| Drug | .013 | 1,16 | .91 | .000 |
| Age x Drug | 2.972 | 1,16 | .093 | .076 |

**Table S7:** Mixed ANOVA results for Experiment 1 degradation instrumental retraining data (dependent measure: baseline-normalized press rate). Degradation (Deg vs. Nondeg) and Day (1-10) varied within-subjects while Age (Adult vs. Adolescent) and Drug (THC vs. vehicle) varied between subjects. See main text for details. Significant results (p < .05) indicated by **bold text**.

| Table S8: Degradation test 1 (normalized to pre-deg baseline) | | | | |
| --- | --- | --- | --- | --- |
| *Early test* | | | | |
|  | F value | d.f. | P value | η_p_^2^ |
| Degradation | **6.13** | **1,36** | **.02** | **.15** |
| Deg x Age | .74 | 1,36 | .40 | .020 |
| Deg x Drug | .51 | 1,36 | .48 | .014 |
| Day x Age x Drug | 1.61 | 1,36 | .21 | .043 |
| Age | .099 | 1,36 | .76 | .003 |
| Drug | .30 | 1,36 | .59 | .008 |
| Age x Drug | .69 | 1,36 | .41 | .019 |
| *Late test* | | | | |
|  | F value | d.f. | P value | η_p_^2^ |
| Degradation | **35.15** | **1,35** | **<.001** | **.501** |
| Deg x Age | .087 | 1,35 | .77 | .002 |
| Deg x Drug | .11 | 1,35 | .75 | .003 |
| Day x Age x Drug | **4.37** | **1,35** | **.044** | **.11** |
| Age | .051 | 1,35 | .82 | .001 |
| Drug | .009 | 1,35 | .93 | .000 |
| Age x Drug | .038 | 1,35 | .85 | .001 |

**Table S8:** Mixed ANOVA results for Experiment 1 degradation instrumental retraining data (separated for Early and Late test sessions; dependent measure: baseline-normalized press rate). Degradation (Deg vs. Nondeg) varied within-subjects while Age (Adult vs. Adolescent) and Drug (THC vs. vehicle) varied between subjects. See main text for details. Significant results (p < .05) indicated by **bold text**.

| Table S9: THC effects on bodyweight | | | | |
| --- | --- | --- | --- | --- |
| *First injection* | | | | |
|  | F value | d.f. | P value | η_p_^2^ |
| **Age** | **1164.82** | **1,36** | **<.001** | **.97** |
| Drug | .03 | 1,36 | .86 | .001 |
| Age x Drug | .13 | 1,36 | .72 | .004 |
| *Last injection* | | | | |
|  | F value | d.f. | P value | η_p_^2^ |
| **Age** | **856.76** | **1,36** | **<.001** | **.96** |
| **Drug** | **6.30** | **1,36** | **.017** | **.15** |
| Age x Drug | .18 | 1,36 | .67 | .005 |
| *Prior to testing* | | | | |
|  | F value | d.f. | P value | η_p_^2^ |
| **Age** | **38.42** | **1,36** | **<.001** | **.52** |
| Drug | .043 | 1,36 | .84 | .001 |
| Age x Drug | .030 | 1,36 | .86 | .001 |

**Table S9:** ANOVA results for Experiment 2 bodyweight data on the first and last injection day and prior to behavioral testing (dependent measure: grams). Age (Adult vs. Adolescent) and Drug (THC vs. vehicle) varied between subjects. See main text for details. Significant results (p < .05) indicated by **bold text**.

| Table S10: SCM consumption testing | | | | |
| --- | --- | --- | --- | --- |
| *3-min bins* | | | | |
|  | F value | d.f. | P value | η_p_^2^ |
| **Bin** | **67.48** | **29,1015** | **<.001** | **.66** |
| **Bin x Age** | **3.55** | **29,1015** | **<.001** | **.092** |
| Bin x Drug | .46 | 29,1015 | .99 | .013 |
| Bin x Age x Drug | 1.06 | 29,1015 | .38 | .029 |
| Age | .052 | 1,35 | .82 | .001 |
| Drug | .16 | 1,35 | .70 | .004 |
| Age x Drug | .59 | 1,35 | .45 | .016 |
| *Early intake* | | | | |
|  | F value | d.f. | P value | η_p_^2^ |
| **Concentration** | **12.84** | **3,105** | **<.001** | **.27** |
| Conc x Age | .87 | 3,105 | .46 | .024 |
| Conc x Drug | .73 | 3,105 | .54 | .020 |
| Conc x Age x Drug | .74 | 3,105 | .52 | .021 |
| **Age** | **7.05** | **1,35** | **.012** | **.17** |
| Drug | .49 | 1,35 | .49 | .014 |
| Age x Drug | .35 | 1,35 | .56 | .010 |
| *Total intake* | | | | |
|  | F value | d.f. | P value | η_p_^2^ |
| **Concentration** | **72.84** | **3,105** | **<.001** | **.68** |
| Conc x Age | .58 | 3,105 | .63 | .016 |
| Conc x Drug | .81 | 3,105 | .49 | .023 |
| Conc x Age x Drug | .49 | 3,105 | .69 | .014 |
| Age | .277 | 1,35 | .60 | .008 |
| Drug | .45 | 1,35 | .51 | .013 |
| Age x Drug | .17 | 1,35 | .69 | .005 |
| *Total beam breaks* | | | | |
|  | F value | d.f. | P value | η_p_^2^ |
| **Concentration** | **6.04** | **3,105** | **<.001** | **.15** |
| Conc x Age | .51 | 3,105 | .67 | .014 |
| Conc x Drug | .76 | 3,105 | .52 | .021 |
| Conc x Age x Drug | .84 | 3,105 | .48 | .023 |
| **Age** | **16.16** | **1,35** | **<.001** | **.32** |
| Drug | .69 | 1,35 | .41 | .019 |
| **Age x Drug** | **7.16** | **1,35** | **.011** | **.17** |

**Table S10:** Mixed ANOVA results for Experiment 2 SCM consumption test data (dependent measure: bodyweight normalized intake (ml/kg) for first three sections and total beam breaks for last section). 3-min bin (1-30) and SCM concentration (5, 10, 25, 50%) varied within-subjects while Age (Adult vs. Adolescent) and Drug (THC vs. vehicle) varied between subjects. See main text for details. Significant results (p < .05) indicated by **bold text**.

| Table S11: Progressive ratio performance | | | | |
| --- | --- | --- | --- | --- |
|  | F value | d.f. | P value | η_p_^2^ |
| **Concentration** | **35.40** | **1,35** | **<.001** | **.50** |
| Con x Age | .059 | 1,35 | .86 | .002 |
| Con x Drug | 1.49 | 1,35 | .23 | .041 |
| Con x Age x Drug | .022 | 1,35 | .88 | .001 |
| Age | .16 | 1,35 | .70 | .004 |
| Drug | 2.13 | 1,35 | .15 | .057 |
| Age x Drug | 1.88 | 1,35 | .18 | .051 |

**Table S11:** Mixed ANOVA results for Experiment 2 progressive ratio performance data (dependent measure: total rewards earned). SCM concentration (5% vs. 50%) varied within-subjects while Age (Adult vs. Adolescent) and Drug (THC vs. vehicle) varied between subjects. See main text for details. Significant results (p < .05) indicated by **bold text**.

| Table S12: Rimonabant effects on progressive ratio performance | | | | |
| --- | --- | --- | --- | --- |
| *Linear trend analysis* | | | | |
|  | F value | d.f. | P value | η_p_^2^ |
| **Dose** | **22.59** | **1,35** | **<.001** | **.39** |
| Dose x Age | .71 | 1,35 | .41 | .020 |
| Dose x Drug | .27 | 1,35 | .61 | .008 |
| Dose x Age x Drug | 3.30 | 1,35 | .078 | .086 |
| *Quadratic trend analysis* | | | | |
|  | F value | d.f. | P value | η_p_^2^ |
| Dose | 3.93 | 1,35 | .055 | .10 |
| Dose x Age | .27 | 1,35 | .61 | .008 |
| Dose x Drug | 1.43 | 1,35 | .24 | .04 |
| Dose x Age x Drug | 3.41 | 1,35 | .073 | .089 |
| *Between-subjects effects* | | | | |
|  | F value | d.f. | P value | η_p_^2^ |
| Age | .42 | 1,35 | .52 | .012 |
| Drug | 3.8 | 1,35 | .059 | .098 |
| Age x Drug | 1.31 | 1,35 | .26 | .036 |

**Table S12:** Mixed ANOVA results for Experiment 2 effects of rimonabant pretreatment on progressive ratio performance data (dependent measure: total rewards earned). Rimonabant dose (0, .3, 1, and 3 mg/kg) varied within-subjects while Age (Adult vs. Adolescent) and Drug (THC vs. vehicle) varied between subjects. Repeated measures analyses focused on linear and quadratic contrasts. See main text for details. Significant results (p < .05) indicated by **bold text**.
